# Epicardial Fat Thickness: A Cardiometabolic Risk Marker in Rheumatoid Arthritis

**DOI:** 10.7759/cureus.21397

**Published:** 2022-01-18

**Authors:** Sekhar Saha, Rajnish Singh, Irfan A Mir, Naman Bansal, Pankaj K Singh, Mir Nadeem

**Affiliations:** 1 Internal Medicine, Postgraduate Institute of Medical Education & Research, Dr. Ram Manohar Lohia Hospital, New Delhi, IND; 2 Internal Medicine, School of Medical Sciences & Research, Greater Noida, IND; 3 Emergency Medicine, School of Medical Sciences & Research, Greater Noida, IND; 4 Internal Medicine, King Khalid University and Associated Hospital, Abha, SAU

**Keywords:** disease duration, disease activity score, high density lipoprotein, cardiovascular risk factor, epicardial fat thickness, rheumatoid arthritis

## Abstract

Background: Rheumatoid arthritis (RA) is a chronic, systemic, inflammatory disorder of unknown etiology which mainly involves synovial joints. However, the corresponding systemic inflammation may result in disorders of multiple other organ systems. Several organs and organ systems are potentially involved in RA, particularly in severe diseases. The organs most involved are the lung, heart, eyes, and nervous system. Extra-articular manifestations of RA may develop even before the onset of arthritis. Emerging epidemiological evidence shows that cardiovascular disease (CVD) accounts for near about 50% of RA-associated death. Epicardial fat thickness (EFT) has recently emerged as a new marker of cardiometabolic risk. Although Epicardial fat (EF) is needed for heart muscle function, given its intrinsic inflammatory status, EFT displays the potential to serve as a therapeutic target in patients with RA.

Objectives: To evaluate EFT using echocardiography in RA patients compared to age and sex-matched control and to find the factors associated with EFT in RA patients.

Materials and methods: This current study was conducted in the Department of Medicine, Postgraduate Institute of Medical Education & Research. The study was conducted from November 2016 to March 2018. Patients with BMI ≥ 30 kg/m^2^, diabetes mellitus, primary hyperlipidemia, and uncontrolled hypertension were particularly excluded. Thirty patients of age and sex-matched controls were also taken for the study. All the patients and controls selected for the study were subjected to detailed history taking and clinical examination. They were subjected to lab investigations and echocardiography. The 30 RA patients included in the study were diagnosed according to the 2010 ACR-EULAR criteria. Disease activity was measured by the disease activity score (DAS28) index.

Results: Group 1 included 30 patients with RA and group 2 included 30 age and sex-matched controls. Pearson correlation analysis was done between EFT and other variables. Only HDL, erythrocyte sedimentation rate (ESR), high-sensitivity C-reactive protein (hS-CRP), rheumatoid factor (RF), anti-cyclic citrullinated peptide (anti-CCP), DAS28, and disease duration were found to have a significant correlation with EFT.

Conclusion: In patients with RA, EFT, left ventricular mass, and diastolic dysfunction are increased in RA patients compared to healthy controls. Out of the conventional CVD risk factors, only HDL was associated with increased EFT in RA patients. In RA patients, DAS28, disease duration, RF, anti-CCP, and markers of inflammation (ESR, hs-CRP) were also associated with increased EFT.

## Introduction

Rheumatoid arthritis (RA) is a chronic, systemic, inflammatory disorder of unknown etiology which mainly involves synovial joints. The estimated prevalence of RA in the community in India is 0.75% [1]. In RA, the subsequent inflammatory changes lead to cartilage and bone destruction. In addition to this, the corresponding systemic inflammation may result in disorders of multiple other organ systems [2]. Several organs and organ systems are potentially involved in RA, particularly in severe diseases. Extra-articular manifestations occur in about 40% of patients with RA over a lifetime of disease [3]. Other than joints, the organs most involved are the lung, heart, eyes, and nervous system. Extra-articular manifestations of RA may develop even before the onset of arthritis. Emerging epidemiological evidence shows that cardiovascular disease (CVD) accounts for near about 50% of RA-associated death. Epicardial fat thickness (EFT) has recently emerged as a new marker of cardiometabolic risk [4]. Although epicardial fat (EF) is needed for heart muscle function, given its intrinsic inflammatory status, EFT displays the potential to serve as a therapeutic target in patients with RA [5].

Although studies on EFT in RA are very few, it is being suggested that EFT is increased in patients with RA and is associated with increased cardiovascular risk. Indian studies on EFT in RA are not available, and to our knowledge, it is the first study on EFT in RA patients. In our study, we aim to evaluate EFT using echocardiography (ECHO) in RA patients compared to age and sex-matched controls and to determine factors that are associated with EFT.

## Materials and methods

This current study was a hospital-based cross-sectional observational study, which was conducted in the Department of Medicine, Postgraduate Institute of Medical Education & Research, Dr. Ram Manohar Lohia Hospital, New Delhi. The study was conducted from November 2016 to March 2018. The total number of patients included in the study was 30 patients. Patients aged > 18 years were selected from the general outpatient department (OPD) and rheumatology clinic run by the department of medicine. Patients included in the study satisfied the inclusion and exclusion criteria and gave their written informed consent. Patients with body mass index (BMI) ≥ 30 kg/m^2^, diabetes mellitus, primary hyperlipidemia, and uncontrolled hypertension were particularly excluded. Thirty patients of age and sex-matched controls were also taken for the study. All the issues including the ethical issues were evaluated by the Institutional Review Board and approved via notification number TP(MD/MS) (61/2016) /IEC/PGIMER/RMLH 7765/16 (Dated: 5/10/2016). All the patients and controls selected for the study were subjected to detailed history taking and clinical examination. They were subjected to lab investigations and ECHO. The 30 RA patients included in the study were diagnosed according to the 2010 American College of Rheumatology-European League Against Rheumatism (ACR-EULAR) criteria [6]. Disease activity was measured by the disease activity score (DAS28) index [7].

ECHO with pulse wave Doppler was performed in Echo Lab at the Department of Cardiology by a single cardiologist who was blind to patient details. A transthoracic two-dimensional (2D) ECHO examination was performed by PHILIPS HD 11XE machine, using the standard technique with study population in the left lateral decubitus position using a 3.5-MHz transducer. All the measurements were taken and interpreted following the American Society of Echocardiography recommendations [8]. The EFT was measured according to the method first described and validated by Iacobellis et al. [9]. The EF was identified as the echo-free space between the outer wall of the myocardium and the visceral layer of the pericardium. EFT was measured in the parasternal long-axis view, perpendicularly on the free wall of the right ventricle at end-systole in three cardiac cycles. Maximum EFT was measured at the point on the free wall of the right ventricle along the midline of the ultrasound beam, perpendicular to the aortic annulus, used as an anatomical landmark for this view. The average value of three cardiac cycles was considered [10].

Left ventricular mass (LVM) was similarly determined using an anatomically validated formula of Devereux et al. [11]:

LVM=0.8{1.04[([LVEDD + IVSD + PWD]3 − LVEDD3)]} + 0.6, where LVEDD, IVSD, and PWD represent left ventricular end-diastolic diameter, interventricular septal diameter, and posterior wall diameter in diastole, respectively, was derived assuming LV dimensions in centimeters. LVEDD, PWD, and IVSD were measured in parasternal long-axis view by M mode.

Left ventricular diastolic dysfunction was diagnosed and graded based on measurements of early (E wave) and late (A wave) diastolic mitral flow velocities, determined on pulsed wave Doppler. Continuous variables were presented as mean ± SD. Categorical variables were presented as the number with percentage (%) in the bracket. Statistical tests were applied as follows:

1. Quantitative variables were compared using an unpaired t-test between the two groups.

2. Qualitative variables were compared using the Chi-square test.

3. Pearson correlation was used to determine the association between two variables.

The data were entered in MS EXCEL spreadsheet and analysis was done using Statistical Package for Social Sciences (SPSS, Chicago, IL, USA) version 18.0. P-value <0.05 was considered statistically significant.

## Results

The study population was divided into two groups for further description and analysis: group 1 included 30 patients with RA and group 2 included 30 age and sex-matched controls.

In Table 1, the two groups were comparable for age, sex, BMI, SBP, DBP, FBS, eGFR, TC, HDL, LDL, and TG. Erythrocyte sedimentation rate (ESR) and FBS were higher in cases compared to controls as expected.

**Table 1:** Comparison of clinical characteristics in two groups Data have been expressed as Mean ± SD unless stated otherwise. *Data expressed as a number with percentage inside bracket.

	Group 1 (N=30)	Group 2 (N=30)	P-value
Age (years)	40.97 ± 7.44	41.07 ± 6.91	0.96
Sex (m/f)*	4(13%) /26 (87%)	1(3%) /29 (97%)	0.161
Body Mass Index (BMI) (kg/m^2^)	23.93 ± 2.73	23.46 ± 2.32	0.47
Systolic Blood Pressure (SBP) (mm of Hg)	120.60 ± 10	120 ± 9.51	0.81
Diastolic Blood Pressure (DBP) (mm of Hg)	79.80 ± 7.07	79.27 ± 6.53	0.76
Fasting Blood Sugar (FBS) (mg/dL)	85.06 ± 7.29	81.97 ± 8.10	0.13
Estimated Glomerular Filtration Rate (eGFR) (mL/min/1.73 m^2^)	86.51 ± 19.10	89.65 ± 25.18	0.59
Total Cholesterol (TC) (mg/dL)	176.80 ± 41.76	168.70 ± 19.20	0.34
Low-Density Lipoprotein (LDL) (mg/dL)	100.17 ± 25	106.47 ± 24.94	0.33
High-Density Lipoprotein (HDL) (mg/dL)	38 ± 9.24	38.77 ± 7.50	0.73
Triglycerides (TG) (mg/dL)	122.17 ± 36.79	135.47 ± 38.70	0.18
Erythrocyte Sedimentation Rate (ESR) (mm)	36.40 ± 22.19	15.90 ± 6.85	<0.001
Disease Duration (years)	4.75 ± 2.21	—	
Disease Activity Score (DAS28)	2.89 ± 0.84	—	
Rheumatoid Factor (RF) (U/mL)	16.90 ± 7.44	—	
Anti-Cyclic Citrullinated Peptide (Anti-CCP) (U/mL)	104.22 ± 92.89	—	
high sensitive C Reactive Protein (hS-CRP) (mg/L)	7.33 ± 2.78	—	

In Table 2, EFT, LVEDD, IVSD, LVM, and LVDD were higher in cases compared to controls. EF, which represents a systolic function, and posterior wall thickness at end-diastole were similar in the two groups.

**Table 2:** Comparison of echocardiographic parameters in two groups Data have been expressed as Mean ± SD unless stated otherwise.

	Group 1 (N=30)	Group 2 (N=30)	P-value
Epicardial Fat Thickness (EFT) (mm)	5.08 ± 0.79	4.33 ± 0.73	<0.001
Left Ventricular End Diastolic Dysfunction (LVEDD) (cm)	4.97 ± 0.67	4.27 ± 0.45	<0.001
Posterior Wall Thickness at end Diastole (PWD) ( cm)	0.87 ± 0.06	0.86 ± 0.05	0.22
Interventricular Septal Diameter (IVSD) (cm)	0.89 ± 0.07	0.85 ± 0.04	0.01
Left Ventricular Mass (LVM) (gm)	153.07 ± 44.00	112.20 ± 23.13	<0.001
Ejection Fraction (EF) (%)	60.67 ± 2.17	61.67 ± 2.40	0.10
Left Ventricular Diastolic Dysfunction (LVDD)	Grade1-8 (26%)	Grade1-4 (13%)	0.02
	Grade2-2 (7%)	Grade2-0 (0%)	0.014

Table 3 represents Pearson correlation analysis between EFT and other variables. Only high-density lipoprotein, ESR, high-sensitivity C-reactive protein (hS-CRP), rheumatoid factor (RF), anti-cyclic citrullinated peptide (anti-CCP), DAS28, and disease duration were found to have a significant correlation with EFT. However, no correlation was found between EFT and other conventional risk factors of CVD like age, sex, BMI, SBP, DBP, FBS, LDL, and triglycerides.

**Table 3:** Correlation of epicardial fat thickness (EFT) with different factors

	R	P-value
Age (years)	0.353	0.056
Sex (m/f)	0.087	0.647
Body Mass Index (BMI) (kg/m^2^)	0.343	0.064
Systolic Blood Pressure (SBP) (mm of Hg)	0.059	0.758
Diastolic Blood Pressure (DBP) (mm of Hg)	0.32	0.084
Fasting Blood Sugar (FBS) (mg/dL)	0.026	0.892
Triglycerides (TG) (mg/dL)	0.288	0.123
High-Density Lipoprotein (HDL) (mg/dL)	-0.687	<0.001
Low-Density Lipoprotein (LDL) (mg/dL)	0.12	0.529
DIsease Duration (years)	0.443	0.014
Erythrocyte Sedimentation Rate (ESR) (mm)	0.511	<0.001
Rheumatoid Factor (RF) (U/mL)	0.470	0.009
Anti-cyclic Citrullinated Peptide (Anti-CCP) (U/mL)	0.413	0.023
high sensitivity C Reactive Protein (hS CRP) (mg/L)	0.392	0.032
Disease Activity Score (DAS28)	0.551	0.002

**Figure 1:**
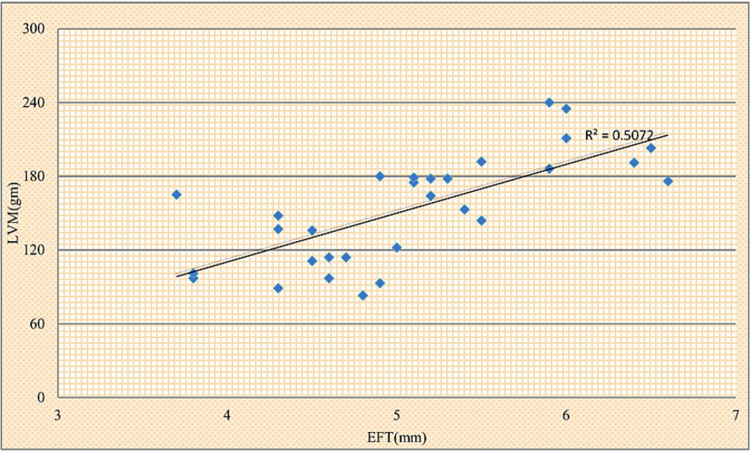
Scattered plot showing correlation of epicardial fat thickness (EFT) with left ventricular mass (LVM). r=0.712, p-value=<0.001

**Figure 2:**
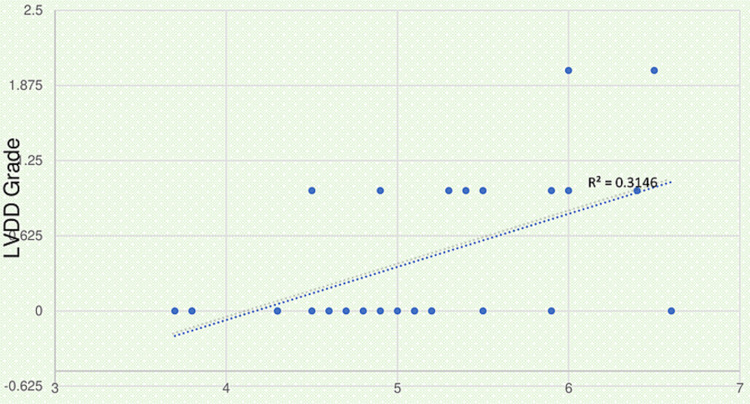
Scattered plot showing correlation of epicardial fat thickness (EFT) with left ventricular diastolic dysfunction (LVDD). r=0.561, p-value=0.001

Figures 1, 2 showed that LVM and LVDD correlate positively with EFT, respectively. 

 

## Discussion

Epicardial adipose tissue (EAT) is a proxy measure for visceral adiposity and visceral fat has been shown to be a reliable predictor of metabolic risk [12]. An increasing body of research suggests that EAT thickness is linked to traditional anthropometric and clinical factors such as BMI, waist circumference (WC), systolic blood pressure, and diastolic blood pressure [13,14]. The goal of this study was to compare EFT in RA patients to age and sex-matched controls, as well as to determine the variables linked to increased EFT in RA patients. The traditional risk factors have failed to explain the higher CVD mortality and morbidity in RA patients, and EFT is becoming more well recognized as a CVD risk.

We tested the study population for EFT, LVM, and LVDD using an ECHO with a color Doppler and also looked for traditional risk variables as well as disease status indicators including RF, anti-CCP, disease duration, DAS28, and inflammatory markers like ESR, hS-CRP. The EFT, LVDD, and LVM were also measured in the controls along with traditional cardiovascular risk variables. In the research group, the average EFT was 5.08 ± 0.79 mm, with ranges ranging from 3.8 mm to 6.5 mm. The average EFT in the control group was 4.33 ± 0.73 mm, with ranges of 3.0 mm to 5.6 mm. When compared to the control group, EFT was found to be considerably greater (p-value 0.001) in the study group (Table 1). Similar findings were obtained in studies by Iacobellis et al. [10], Natale et al. [15], Nelson et al. [16], and Lima-Martnez et al. [17]. Our study is the first of its kind in the Asian subcontinent with no studies in India so far.

In our study, we went a step further and used ECHO and Doppler to evaluate LVM in both groups, found that the average LVM in the study group was 153.07 ± 44.00 g and in the control group was 112.20 ± 23.13 g, which was statistically significant (Table 2). Hence, we discovered that patients with RA had higher LVM than age and sex-matched controls and that when dispersed diagrams with EFT were drawn, EFT rose considerably and proportionally with LVM (Figure 1). This is consistent with the findings of Rudominer et al. [18] and Avina-Zubieta et al. [19].

In addition, 26% of patients in the study group developed grade one LVDD, compared to 13% in the control group (p-value 0.02). In addition, 7% of patients in the study group had grade two LVDD, whereas no one in the control group had grade two LVDD (p-value- 0.014). According to research conducted by Liang et al. [20], the prevalence of LVDD in patients with RA is high. They discovered that RA patients had a greater incidence of LVDD than healthy controls and that RA duration is also linked to LVDD. The LVDD has been seen in people with RA who do not have clinically significant heart dysfunction in previous research.

The age, sex, BMI, fasting blood sugar, lipid profile, blood pressure, and estimated glomerular filtration rate were compared and statistical analysis was performed between the two groups. Among them, FBS and ESR were determined to have significant differences between these two groups. Only HDL was linked to greater EFT in RA patients of the traditional CVD risk variables. Higher EFT was also linked to increased DAS28, disease duration, RF, anti-CCP, and inflammatory markers (ESR, hs-CRP) in RA patients (Table 3).

In the study group, Pearson correlation had been done between EFT as a dependant variable with conventional risk factors and markers of disease activity and markers of inflammations as independent variables in cases. It was discovered that EFT has a strong relationship with HDL (r-0.687, p-0.001), ESR (r-0.511, p-0.001), hS-CRP (r-0.392, p-0.032), RF (r-0.470, p-0.009), anti-CCP (r-0.413, p-0.023), disease duration (r-0.443, p-0.014), DAS 28 (r-0.551, p-0.002) Table 3, LVM (r-0.712, p-<0.001) (Figure 1), and LVDD (r-0.561, p-0.001) (Figure 2).

Limitations

The primary limitation of our study was the small sample size. A small sample size has low statistical power and thus may yield false-negative results. The other limitation of our study it was a cross-sectional design, and the results cannot be generalized to the general population. Neither can we apply our results to the general population due to the numerous exclusion criteria?

Despite this, we believe that our findings provide a valuable contribution to the EFT and RA. Hence, future bigger and prospective studies are required to confirm our results.

## Conclusions

In patients with RA, EFT, LVM, and diastolic dysfunction are increased in RA patients compared to healthy controls. The increased EFT is correlated positively with independent CVD risk factors like increased LVM and LVDD. Out of the conventional CVD risk factors, only HDL was associated with increased EFT in RA patients.

In RA patients, DAS28, disease duration, RF, anti-CCP, and markers of inflammation (ESR, hs-CRP) were also associated with increased EFT.
